# Anti-angiogenic effect of high doses of ascorbic acid

**DOI:** 10.1186/1479-5876-6-50

**Published:** 2008-09-12

**Authors:** Nina A Mikirova, Thomas E Ichim, Neil H Riordan

**Affiliations:** 1 Bio-Communications Research Institute, Wichita, Kansas, USA; 2 Medistem Laboratories Inc, Chandler, Arizona, USA

## Abstract

Pharmaceutical doses of ascorbic acid (AA, vitamin C, or its salts) have been reported to exert anticancer activity *in vitro* and *in vivo*. One proposed mechanism involves direct cytotoxicity mediated by accumulation of ascorbic acid radicals and hydrogen peroxide in the extracellular environment of tumor cells. However, therapeutic effects have been reported at concentrations insufficient to induce direct tumor cell death. We hypothesized that AA may exert anti-angiogenic effects. To test this, we expanded endothelial progenitor cells (EPCs) from peripheral blood and assessed, whether or not high dose AA would inhibit EPC ability to migrate, change energy metabolism, and tube formation ability. We also evaluated the effects of high dose AA on angiogenic activities of HUVECs (human umbilical vein endothelial cells) and HUAECs (human umbilical arterial endothelial cells). According to our data, concentrations of AA higher than 100 mg/dl suppressed capillary-like tube formation on Matrigel for all cells tested and the effect was more pronounced for progenitor cells in comparison with mature cells. Co-culture of differentiated endothelial cells with progenitor cells showed that there was incorporation of EPCs in vessels formed by HUVECs and HUAECs. Cell migration was assessed using an *in vitro* wound healing model. The results of these experiments showed an inverse correlation between AA concentrations relative to both cell migration and gap filling capacity. Suppression of NO (nitric oxide) generation appeared to be one of the mechanisms by which AA mediated angiostatic effects. This study supports further investigation into non-cytotoxic antitumor activities of AA.

## Background

The anti-cancer mechanism of high dose AA has been reviewed in numerous papers [review in papers [1,2]]. The mechanism by which high-dose AA induces cytotoxicity of tumor cells remains controversial. The most common theory of ascorbic acid tumor toxicity relates to its oxidation-reduction properties. In the presence of oxygen, AA undergoes spontaneous oxidation, giving rise to dehydroascorbic acid and the superoxide [3-7]. However, as it was shown in studies [8,9], the cytotoxicity of AA to tumor cells depends on the culture medium. Our research [10] documents both *in vitro* and *in vivo* evidence that plasma provides antioxidant protection against reactive oxygen species (ROS) and hydrogen peroxide (H_2_O_2_) formed when 15–50 grams of AA were administered intravenously. Based on studies, which support that high-dose ascorbic acid is cytotoxic to tumor cells, high-dose intravenous ascorbic acid has been applied as cancer therapy. Case reports describing responses of cancer patients to high-dose intravenous vitamin C were reported [11-18]. These reports include several cases of progressive malignant disease having significant partial responses and complete responses to high-dose ascorbic acid as monotherapy. Based on data showing a tumor-cytoprotective effect of plasma and serum products at concentrations of AA that have clinically induced significant regressions in cancer patients, we hypothesized that there may be another anti-tumor action of AA associated with inhibition of angiogenesis. We subsequently analyzed the effect of high concentrations of ascorbic acid (100 mg/dl–300 mg/dl) on *in vitro* endothelial cells and new blood vessel formation.

Angiogenesis is a normal process, required for normal tissue repair and growth. Pathological angiogenesis is characterized by the persistent proliferation of endothelial cells and blood vessel formation. This complex process plays an important role in tumor growth, invasion, and metastasis. Recent studies have linked the involvement of circulating endothelial precursor cells (EPCs) to pathologic angiogenesis [19-27]. Tumor cells signaling vascular proliferation induce endothelial phenotypic expression of the bone marrow progenitor cells. Many tumors are associated with extensive bone marrow-derived cell infiltration, and the role of different subsets of bone marrow-derived cells in tumor development, progression, and metastasis was shown in studies [28-32].

There have been conflicting results reported from studies evaluating the effect of AA on angiogenesis during tumor development. The effect of low concentration of AA (scorbutic) obtained from dietary concentration was analyzed for tumor development in an animal [33]. The absolute number of blood vessels was reduced in ascorbic acid depleted tumors compared to the fully supplemented animals. In contrast, another group found tumor angiogenesis to be independent of collagen synthesis and scorbutic levels of ascorbic acid [34]. In this study, no difference in tumor growth was detected between the ascorbic acid depleted tumors and the fully supplemented ascorbic acid mouse group. Conversely, high concentration of ascorbic acid administered to cauterized corneas was found to suppression of angiogenesis in a rat model [35].

Here, we propose that the high concentrations of ascorbic acid achieved after intravenous administration of 25–60 grams of AA affect both endothelial progenitor cells and mature endothelial cell functions involved in the process of angiogenesis. Evidence supporting this hypothesis will be established from several lines of experimental investigations.

1. The effect of high concentrations of AA on EPCs and mature endothelial cells to migrate, to engage in energy metabolism, and to form capillary tubes.

2. The effect of high concentrations of AA on the decreased production and availability of nitric oxide within endothelial cells resulting in suppressed angiogenesis.

## Methods

### Cell lines

HUVECs and HUAECs were obtained from Cascade Biologics and Cambrex Company. HUVECs were grown in medium M-200 (Cascade Biologics) supplemented by 2% fetal bovine serum (FBS), hydrocortisone, human epidermal growth factor, basic fibroblast growth factor, and heparin. HUAECs were grown in culture basal medium (EGM Bullet Kit, Cambrex), supplemented with bovine brain extract, human endothelial growth factor, hydrocortisone, gentamicin, and 2% fetal bovine serum. Endothelial progenitor cells isolated from peripheral blood were grown in culture with basal medium (EBM-2, Cambrex). All cell lines were grown in 37C and 5% CO_2_.

### Separation of endothelial progenitor cells

Endothelial progenitor cells were separated from adult peripheral blood of several subjects. PBMCs (peripheral blood mononuclear cells) were seeded into 6 well fibronectin coated flasks containing EBM-2 medium. EBM-2 medium was additionally supplemented with growth factors: endothelial growth factor (EGF) and vascular endothelial growth factor (VEGF) with a concentration of 10 ng/ml. Floating cells were discarded after 4 days. The medium was replenished every 3–4 days. Colonies formation began after 10–12 days of incubation.

### Immunofluorescence studies

Cells were detached from plates by Trypsin-EDTA; then washed in PBS containing 2% heat inactivated FBS, and subsequently incubated for another 15 min with serum to block nonspecific sites. Cells were then incubated for another 15 min with either appropriate antibodies or with the relevant control in PBS with 2% FBS.

### Endothelial tube formation assay

96 well plates were coated with 70 ul per well of Matrigel basement membrane matrix (BD Biosciences). Plates were allowed to polymerize at room temperature for 30 min. The cells previously grown in culture were then detached, and 0.02–0.04 M cells resuspended in 100 ul of endothelial basal medium were plated on Matrigel. The plates were examined for tube formation at incubation time references: 3 hrs, 6 hrs and 24 hrs. Each experimental condition was performed in triplicate and repeated several times to assure quality control. Images of each well were captured using the ProRes camera system. For each well image captured, the number of closed loops formed by capillary tubes network was counted by AlphaEase software (Alpha Innotech).

### Nitric oxide production assay

NO production was measured by using DAF-FM diacetate, a specific fluorescence probe for nitric oxide detection (Invitrogen). DAF-FM diacetate is a membrane-permeable dye that is hydrolyzed inside the cells by cytosolic esterases releasing DAF-FM. In the presence of nitric oxide, DAF-FM converts into a fluorescent product, (benzotriazole derivative) which can be detected by fluorometer or flow cytometer. For NO detection, cells were incubated in PBS with 10 mM glucose containing 5 μM DAF-FM-DA for 30 min at 37°C. After the incubation, cells were washed and incubated in the presence of either: inhibitors, stimulators, or ascorbic acid. For endothelial nitric oxide synthase inhibition, a derivative of L-arginine N-nitro-L-arginine methyl ester (L-NAME) was used, and for stimulation of nitric oxide production VEGF was added to medium. Fluorescence was measured by flow-cytometer (Beckman Coulter) and fluorometer (SPEX) at excitation wavelength 490 nm and maximum emission at 514 nm. All measurements of fluorescence were corrected by subtracting the nonspecific fluorescence in medium without addition of dye and in medium with dye but without cells.

### Cell migration assay

Cells migration assay was assessed by the wound healing method as described in [36]. One million cells were seeded in a 35 mm dish with 2 ml of EBM-2. After cells reached confluence, a linear wound was made by scratching the bottom of the dish with a sterile plastic scraper and different concentrations of AA were added in different dishes. The width of the gap was measured by ProgRes imaging system after different time of exposure to AA.

### Method of ATP measurements in cells

Levels of ATP in cells were determined by the CellTiter-GLO Luminescent Cell Viability Assay Kit (Promega Company). This assay generates a luminescence glow type signal produced by a luciferase reaction, and is proportional to the amount of ATP present in the cells. The amount of ATP produced was determined from a standard curve by measuring the level of luminescence for different concentrations of pure ATP (Sigma).

## Results

### 1. Isolation and characterization of the endothelial progenitor cells from adult peripheral blood

To separate endothelial progenitor cells from adult peripheral blood, we used a standard long-time culture protocol [37,38]. Isolation of EPCs from the mononuclear peripheral blood resulted in cobblestone colony appearance of EPCs in culture. The morphology of the cells changed with passages, becoming more elongated cells. All populations of cells were characterized by their surface marker expression and population doubling times. Separated EPCs were positive for CD34, VEGFR2, CD31, CD146, CD144-VE-cadherin, CD105, CD90, and lost CD133. Cells that were used for experiments had fewer than four population doublings.

Endothelial surface markers were compared for mature HUVECs and endothelial progenitor cells. Our research revealed the following data: the markers of mature endothelial cells (CD31, CD146, VEGF-R2/KDR and lectin Ulex europaeus binding) were expressed stronger on HUVECs and less on progenitor cells. HLA-ABC was higher expressed on more committed cells than on less differentiated cells. EPCs were negative for peripheral blood cells markers.

Next, we compared progenitor cells to mature endothelial cells based on their uptake of acetylated low-density lipoprotein (Ac-LDL). Dil-Ac-LDL enters the cells, becomes degraded by lysosomes and subsequently accumulates in the lysosomal membranes. Uptake of acetylated low-density lipoprotein was measured after incubation of cells with 10 ug/ml of Dil-Ac-LDL at 37C in endothelial media for 2 h. According to our data, mature endothelial cells internalized and degraded 2 times more LDL than EPCs.

The third comparison of EPCs to mature endothelial cells was based on these cells ability to make nitric oxide, a substance required to stimulate angiogenesis. The level of NO production was compared in three different state of endothelial cell differentiation: highly proliferative EPCs, low proliferative EPCs (more committed progenitor cells) and mature endothelial cells. The level of fluorescence emission was two times higher in committed endothelial cells and 3–4 times higher in mature endothelial cells in comparison with less committed endothelial progenitor cells. These data suggested that less differentiated cells have a lower level of nitric oxide production or, probably, less expression of endothelial nitric oxide synthase gene. Isolated EPCs were used *in vitro* assays to analyze the level of incorporation of these cells in forming capillary tubes and to determine the effects of the high concentrations of ascorbic acid on energy metabolism and capillary tube formation.

### 2. Effects of high dose ascorbic acid on angiogenesis

The effect of ascorbic acid on capillary tube formation was analyzed for varying high concentrations of AA. In humans, these high-concentrations of AA can be achieved only by intravenous administration of AA. The pharmacokinetics of high concentrations of AA has been summarized in research paper [11]. Pharmacokinetics curves relating high-concentrations of AA (post intravenous administration of 15 g, 30 g, and 60 g) and time of exposure were established. The infusion of 15 g of ascorbic acid in 45 min raised the plasma level of AA to 120 mg/dl with a decrease to half intensity after 2 hours. A 30 g infusion during 80 min increased the maximum level of AA in plasma to 180 mg/dl with elevation of the plasma level above 100 mg/dl during 2.5 hours. While 60 g infused in 80 min resulted in a concentration of AA in blood about 300 mg/dl with duration of intensity of half peak during 2.5 hours. According to these data, the concentrations that were used to analyze the effect of AA on angiogenesis were 50–300 mg/dl with the duration of exposure 3 hours.

**Figure 1:**
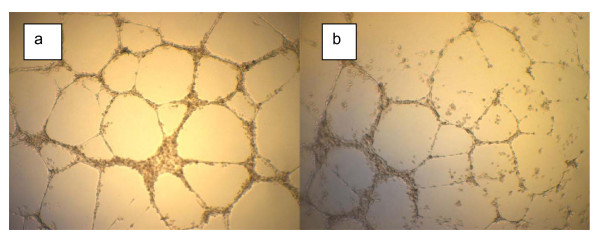
**Effect of high doses of ascorbic acid on capillary tube formation by endothelial progenitor cells**. Formation of capillary tube structure by EPCs in control well (a) and in well treated by 3 mg/ml of ascorbic acid (b).

**Figure 2:**
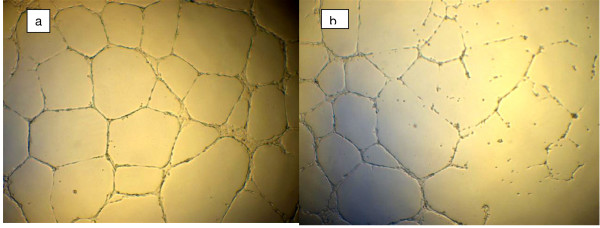
**Effect of high doses of ascorbic acid on capillary tube formation by mature endothelial cells**. Capillary tube formation by HUVECs in control well without addition of ascorbate (a) and in well treated by 3 mg/ml of ascorbic acid (b).

To prove that AA has an effect on endothelial tube formation capacity, we used *in vitro* assays of capillary tube formation on Matrigel. Experiments were performed for several concentrations of serum in medium (2%–100%). AA was added to the culture well at the time of cell plating. Formation of tube vessels started after 1 hour of incubation while tube vessel formation with capillary loops were seen after 3 hours of incubation. This occurred for all endothelial cell lines used: HUVECs; HUAECs; and EPCs. However, as the AA concentration increased past the 50–100 mg/ml point, the number of capillary loops formed began to decrease in number for all cell lines (Figures 1, 2). Figure 1 shows the effect of high doses of ascorbic acid on capillary formation by endothelial progenitor cells. The images are presented for control well (a) and well with cells treated by 300 mg/dl of ascorbic acid (b). Effect of high doses of ascorbic acid on tube formation by mature endothelial cells is shown in Figure 2 for control well (a) and well with 300 mg/dl ascorbic acid added.

**Figure 3:**
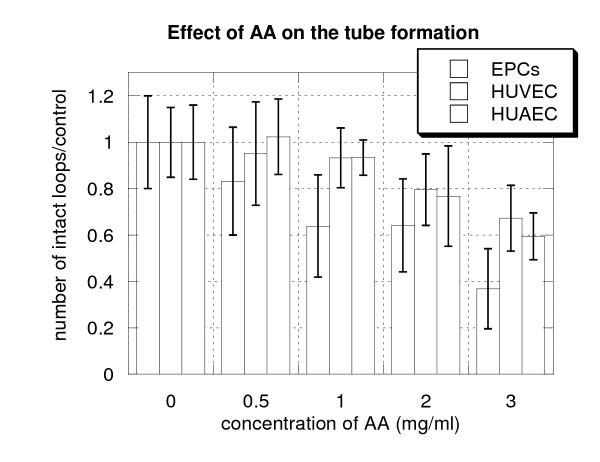
**Ascorbic acid attenuates tube formation in HUVECs, HUAECs and EPCs**. Average data for three cell lines treated by different concentrations of AA during 3–6 hrs. Number of intact loops in wells treated by ascorbic acid was normalized on the number of intact loops in control wells.

The average data for all experiments conducted for all three cell lines are presented in Figure 3. Data used for Figure 3 were collected after 3–6 hours of culture medium exposure for both endothelial progenitor cells and mature endothelila cells to the varied AA concentrations used. Data were averaged for each concentration of AA, and the number of closed loops was normalized on the number of intact closed loops in control wells.

According to these data, formation of vascular structure was significantly reduced for EPCs and mature endothelial cells when AA exceeded concentration 100 mg/dl. The inhibitory effect for EPCs was greater than for mature endothelial cells. Very few closed tube loops were remained in wells growing EPCs when the concentrations of AA reached 200–300 mg/dl of AA. These data suggest that higher concentrations of AA (greater than 100 mg/dl) suppress capillary-like tube formation and angiogenesis.

To find the effect of the same concentrations of AA on existing vessels, we performed experiments with mature endothelial cells. HUVECs and HUAECs cells were preplated and a tube network was established during a 24 h period. After 24 h of incubation of the cells on Matrigel, ascorbic acid was added to the culture wells. The number of closed vessel loops were counted and compared before and after AA exposure. The results did not show a significant difference between the number of intact tubes and closed loops for control wells, wells with low concentrations of AA (10–50 mg/dl), and wells with high concentrations of AA (100–300 mg/dl).

### 3. Effect of co-incubation of endothelial projenitor cells and HUVECs on capillary formation

To estimate the contribution of EPCs in vessel formation, when EPCs and HUVECs are co-incubated, we prepared the Martigel culture wells in two different ways: (1) optimal cell density plating using the same concentration of cells, or (2) plating the wells with half of each cell population. Differentiated endothelial cells plated with the same concentrations as EPCs formed more developed structure with increased number of closed loops. The presence of the EPCs increased the number of closed loops, but the sum of the cells did produce the same count of vessels.

The addition of EPCs increased the number of intact tubes on 40–50% from expected value. However, co-culture of differentiated cells with progenitor cells showed the incorporation of EPCs in blood vessels. These results indicate that EPCs facilitate tubule formation and integrated into the angiogenic structure, but another mechanism of cell-cell interaction by secretion of cytokines and growth factors by EPCs must be analyzed.

### 4. Effect of high doses of AA on migration of endothelial cells

**Figure 4:**
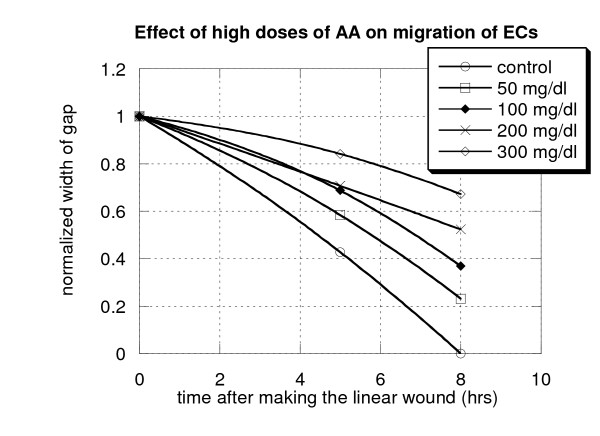
**Effect of high doses of ascorbic acid on endothelial cell migration**. Wound was created by sterile plastic scraper and width of gap was measured after 5 hrs and 8 hrs. The ability of cell migration was calculated as the ratio of the gaps after five and eight hours of the cells' treatment by different concentrations of AA to the initial width of the gap.

Cells migration assay was assessed by the wound healing method as described in Methods. The width of the gap was measured at: 3 hrs; 5 hrs; 8 hrs; and 24 hrs past time the AA was added to the dishes. For each time of measurement, the size of gap was estimated for several different positions, and data were averaged. Data in Figure 4 depicts the ratio of the gap after five and eight hours of the cells' treatment by different concentrations of AA and before addition of AA. The results indicate the differences in both cell migration and gap filling capacities in response to different concentrations of AA. The control wells (without supplementation by AA) showed the cells completing the gap filling within 8 hours. In wells where cells were exposed to high concentrations of AA (300 mg/dl) only 30% of the gap was filled within 8 hours. In wells, where the cells received 100–200 mg/dl of ascorbic acid, endothelial cells demonstrated decreased migration potential with gap filling expressed at only 50%–60% at 8 hours.

To prove that the difference in gap filling was due to migration of endothelial cells and not due to cell proliferation, we measured the level of cell proliferation for the same concentrations of ascorbic acid during the same time of exposure. Proliferation was measured by ATP assay. These studies demonstrated that exposure of cells to 10–50 mg/dl of AA during 3–5 h period did not change energy metabolism of cells or number of cells. The level of metabolic activity was decreased on 20% for concentrations of AA 100–300 mg/dl, but there was no loss of the cells' viability.

These experiments proved that ascorbic acid at high concentration could affect endothelial cells migration. Inhibiting endothelial cell migration is one process of limiting tumor angiogenesis in cancer patients.

### 5. Effects of nitric oxide inhibitor on angiogenesis and high doses of AA on the level of nitric oxide production

To explore a possible mechanism by which high doses of AA may affect angiogenesis, we analyzed the effect of nitric oxide on the process of angiogenesis and the effect of high doses of AA on the level of NO in endothelial cells. In the last two decades, nitric oxide has been shown to promote angiogenesis and vasculogenesis [39]. NO is also an important modulator for the expression of endogenous angiogenic factors such as VEGF and basic FGF [40]. Further, NO has been shown to be involved in tumor angiogenesis [41-44]. Tumors that generate NO constantly have a significantly more developed vascular network and are more invasive [45]. As the result, angiogenesis is dependent of the level of nitric oxide, which has an effect on the migration and specific motivity of the endothelial cells [46].

**Figure 5:**
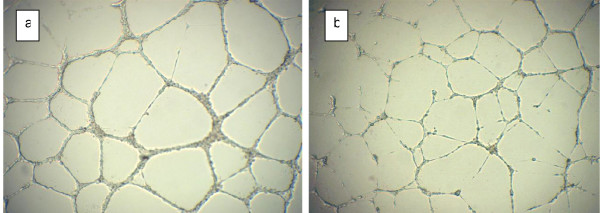
**Effect of NOS inhibitor L-NAME on capillary formation by endothelial cells**. Comparison of the capillary tube structure for endothelial cells treated by 2 mM of nitric oxide synthase inhibitor (b) with control well (a).

**Figure 6:**
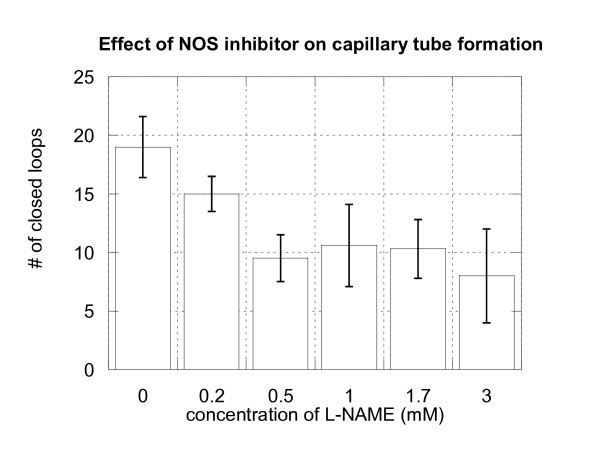
**Nitric oxide inhibitor attenuates formation of capillary network on Matrigel by endothelial cells**. Dependence of the number of closed loops formed by HUVECs on the concentration of NO inhibitor.

The next study was prepared to determine if nitric oxide inhibition could decrease the process of angiogenesis. To find the effect of NO inhibition on angiogenesis, cells incubated on Matrigel were exposed to L-NAME with concentrations 0.2–3 mM. Images of capillary type vessels were made after 24 h. An example of capillary tube formation in a control well and in a well with addition of 2 mM L-NAME is shown in Figure 5. Reduction of the formation of capillary-like structure by HUVECs and HUAECs cells after treatment by different concentrations of L-NAME is shown in Figure 6. The addition of L-NAME to medium with endothelial cells caused a dose dependent inhibition of angiogenesis, which ranged from 16% for 0.2 mM of reagent to 45% for 0.5–3 mM L-NAME. These data strongly suggest that NO formation is an important regulator of the angiogenic process. Use of a NOS inhibitor (L-NAME) markedly decreased the number of capillary tubes formed, thus decreasing angiogenesis.

We then asked the study question: could high concentrations of AA affect nitric oxide production? As the formation of NO appeared to be an important determinant for angiogenesis, we analyzed the effect of high doses of AA on the level of NO production. The level of NO production was measured by using DAF-FM diacetate as described in the Methods. After dye was loaded in the cells, cells were washed twice and incubated with different concentrations of AA. Fluorescence intensity was measured in cells and in supernatant. The results of these measurements demonstrated a decreased levels of NO on 15% ± 8% for concentrations of AA 100 mg/dl, on 23% ± 7% for concentrations of AA 200 mg/dl, and on 30% ± 5% for concentrations of AA 300 mg/dl. Thus a dose dependent decreased production of NO was seen with increasing ascorbic acid concentrations.

## Conclusion

The goal of the present study was to determine the effects of the high doses of AA on process of angiogenesis. Angiogenesis is the process of new blood vessel formation occurring in both normal and cancerous tissues. To make new blood vessels, endothelial cells must migrate toward the angiogenic stimulus, which was released from tumor cells. Endothelial cells must proliferate to provide the necessary number of cells for making new vessels and to form a three-dimensional tubular structure. In addition, circulating endothelial progenitor cells are involved in the development of vasculature, and many tumors are associated with bone marrow-derived endothelial cell infiltration.

According to our study, each of these processes is influenced by high concentration of ascorbic acid: (1) High concentrations of AA alter the metabolic activity of endothelial cells by decreasing the ATP levels by 20% at 300 mg/dl concentration. This prevents significant cell proliferation without changing cell viability. (2) Cell migration: as measured by wound healing assay is decreased by high concentrations of AA. Cell migration was decreased 1.4 times for 200 mg/dl; and 2.4 times for 300 mg/dl. (3) New blood vessel formation: this was measured by *in vitro* endothelial tube formation assay on Matrigel. The effect of AA on angiogenesis estimated by tube formation assay demonstrated inhibitions of vessel structure after 3 h–24 h of exposure of the cells to ascorbic acid. This appeared secondary to AA inhibition of NO in endothelial cells. NO is known as a major stimulus of new blood vessel formation. Our study measured the level of nitric oxide in response to high concentrations of AA. High concentrations of AA inhibited the production of NO, and as NO pathways are important promoters of tumor angiogenesis, high concentrations of AA have been demonstrated to limit angiogenesis.

The decreasing the availability of NO at high concentrations of AA may be explained by the following mechanisms. As endothelial NO formation depends on the presence of intracellular cofactors such as: NADPH, FAD, FMN and tetrahydrobiopterin (BH4), we can suggest that overloading of AA and DHA in cells can change the oxidative-reduction status inside the cells. This could decrease the availability of nitric oxide, through the formation of peroxynitrite. NO can move very rapidly through membranes, thereby the reactions of inactivation may also occur in the extracellular space between cells. Low concentrations of ascorbic acid protect NO from inactivation by superoxide anion and other radicals. High concentrations of ascorbic acid increase the availability of ascorbic acid radicals, resulting in reaction of ascorbic radical with NO. In addition, oxidation of tetrahydrobiopterin, which is a cofactor for endothelial NOS, may affect the availability or the affinity of this factor for nitric oxide production.

Our studies have demonstrated that high concentrations of AA affect the initial phase of cell migration and tube vessel formation and thereby can inhibit angiogenesis.

## Competing interests

The authors declare that they have no competing interests.

## Authors' contributions

NM performed tissue culture experiments, flow cytometric analysis and analysis of data. NR and TI provided input on experimental design and writing of the manuscript. All authors read and approved the final manuscript.
